# Vibration response difference of caving mechanism under coal rock impact based on mechanical–hydraulic coupling

**DOI:** 10.1038/s41598-023-40967-z

**Published:** 2023-08-23

**Authors:** Yanpeng Zhu, Qingliang Zeng, Lirong Wan, Yang Yang, Zhe Li

**Affiliations:** 1 College of Mechanical and Electrical Engineering, Shandong University of Science and Technology, Qingdao, 266590 China; 2 College of Information Science and Engineering, Shandong Normal University, Jinan, 250358 China

**Keywords:** Energy science and technology, Fossil fuels, Engineering, Mechanical engineering

## Abstract

Top coal caving in fully mechanized caving mining will cause an irregular impact on the caving mechanism of hydraulic support. The vibration response of the caving mechanism varies under different forms of impact. This response difference is a prerequisite for new coal rock identification technology in intelligent mining. Therefore, this work studies the difference in vibration response of the caving mechanism under different forms of impact. An innovative mechanical–hydraulic coupling system model of the caving mechanism impact by coal rock is established. The metal plate impact test proved the significant difference in vibration response of the caving mechanism under coal rock impact of different materials. Afterward, a more improved mechanical–hydraulic co-simulation model analyzed the difference in the vibration response of the caving mechanism under different rock materials, volumes, velocities and impact positions. The results show that the vibration response is more intense under rock impact than under coal impact. A lower position, a faster velocity and a larger volume correspond to a more noticeable response difference in the caving mechanism. The vibration and fault sensitive areas of the caving mechanism are determined. This study has a reference significance for improving the caving mechanism's structural design and failure prevention. The conclusions provide guidance for a new intelligent coal rock identification technology based on vibration signals.

## Introduction

Fully mechanized top coal caving mining is developing towards high efficiency, safety, environmental protection and intelligence. The optimization of mining equipment is the critical link to promoting the progress of intelligent technology^1^. The top coal caving hydraulic support is the most crucial supporting equipment in mining, and the caving mechanism is its important control component, which plays a decisive role in the caving effect. Therefore, improving the caving mechanism's structural design and failure prevention is essential.

The top coal caving hydraulic support is developed based on traditional hydraulic support. It has the essential functions of controlling the main roof, maintaining the immediate roof, and pushing the conveyor. In addition, it crushes and caves the top coal. Many scholars have performed innovative research, optimization, and improvement on the top coal caving hydraulic support in recent years. Arasteh et al.^2^ studied the roof caving load of sublevel caving hydraulic support based on a discrete fracture network and cohesive particle model and optimized the bearing performance of the hydraulic support. Zhang et al.^3^ introduced the constitutive model of the top coal caving hydraulic support into CDEM to analyze the coal caving mechanism. Ji et al.^4^ analyzed the anti-overturning, anti-sliding and anti-rotation abilities of the support and improved the anti-slip performance of the support. Through the multi-seam UDEC model, Zhang et al.^5^ studied the influence of the critical layer location on the working resistance of the top coal caving hydraulic support and derived the formula to calculate the maximum support working resistance. In addition, scholars have researched hydraulic supports in complex coal seams, such as large inclination hydraulic supports, light hydraulic supports for thin seams, and large mining height hydraulic supports for thick seams^6–9^.

**Figure 1:**
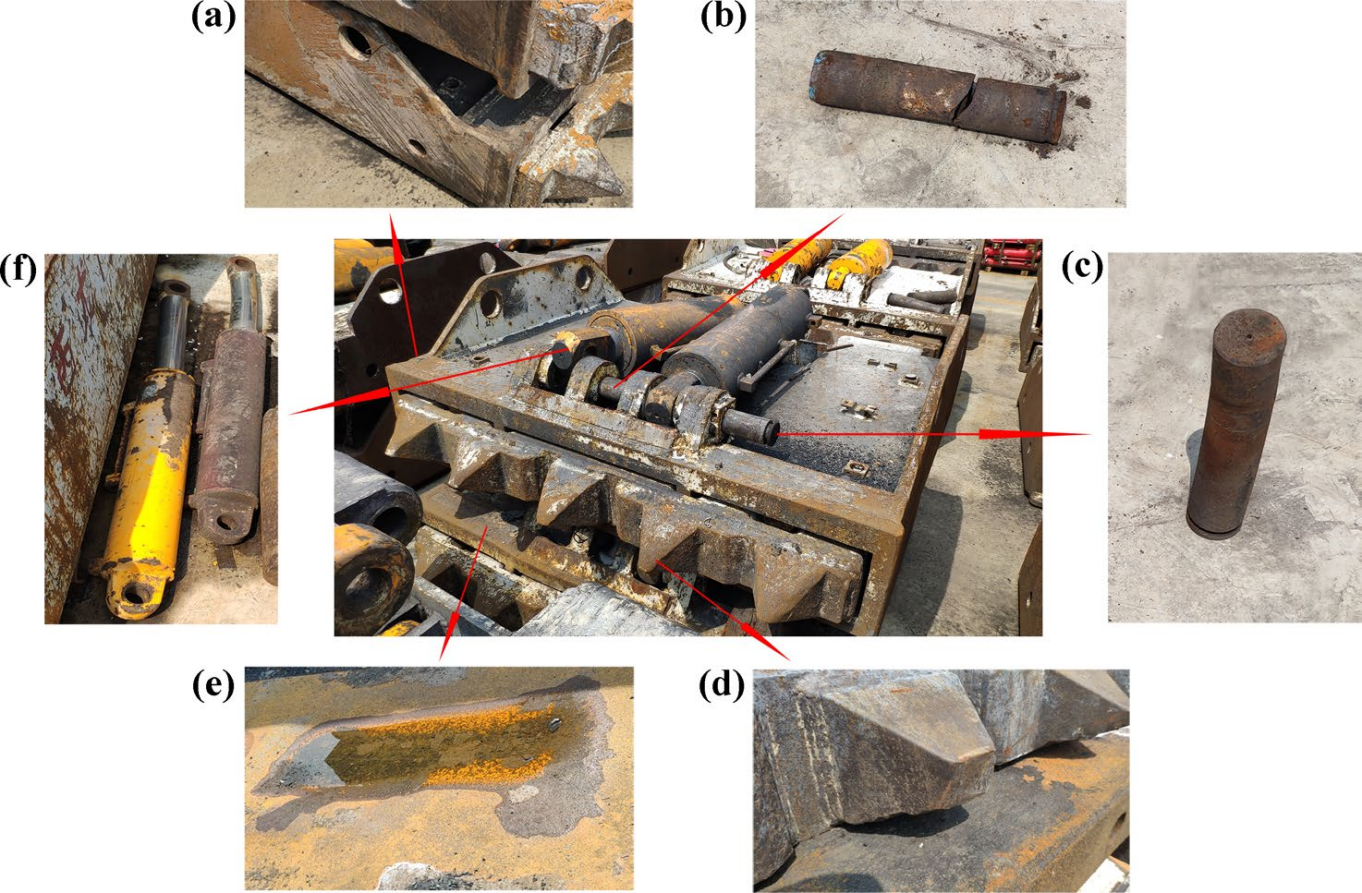
Different failure types of the caving mechanism: (**a**) surface abrasion, (**b**) fracture of the pin shaft, (**c**) bend of the pin shaft, (**d**) tooth wear, (**e**) impact crater of the top surface, and (**f**) bend of jack.

The coal roof fall will cause an impact load on the caving mechanism. When it exceeds the limit, the caving mechanism will be damaged and become invalid, as shown in Fig. 1. Mechanism failure affects the effect of top coal caving and even threatens the safety of workers. Many scholars have performed innovative and optimized research on the caving mechanism. Balasubrahmanyam et al.^10^ developed and verified the first top coal caving distance applicable to geological and mining conditions in India. Zhang et al.^3^ analyzed the caving mechanism of top coal and proposed an improved four-step caving technology. Yang et al.^11^ designed a deep neural network machine learning method to control the coal caving window effectively. Zhang et al.^12^ installed a radar scanning device on the top of the hydraulic support to monitor the top coal thickness in real-time and fed back the signal to the electrohydraulic control system, which realized the intelligent control of the caving mechanism. In addition, scholars have made many innovative improvements to the relevant accessories of the caving mechanism, such as high-precision attitude detection equipment, coal-caving laser scanners, and networked intelligent induction control components^13–15^.

It can be seen that many scholars have studied various aspects of the caving mechanism. These studies include both mechanical structures and hydraulic power systems. However, most of the existing studies focus on a single component or a single function of the caving mechanism, and seldom consider the interaction between the mechanical structure and the hydraulic system. The working environment of the caving mechanism is harsh and complex. The analysis results obtained only from a single angle of mechanical structure or hydraulic system will significantly differ from the actual results. The caving mechanism is a complex mechanism that combines hydraulic systems and machinery. The research from a single perspective is one-sided and hardly reflects its more real mechanical characteristics. The new bidirectional mechanical–hydraulic co-simulation method^16–18^ can precisely establish the relationship between mechanical structure and hydraulic system and more accurately and comprehensively analyze the caving mechanism. Using bidirectional mechanical–hydraulic co-simulation is a complex but essential step in the in-depth study of the caving mechanism. Besides, the response of different areas of the caving mechanism after impact differs. The impact vibration caused by coal and gangue is also different. However, few studies have considered the difference in impact response. This difference in vibration response will also lead to different failure probabilities in different areas of the mechanism^19^. Therefore, on the basis of the feasibility verified by the test, an innovative mechanical–hydraulic co-simulation model of the mining mechanism is established, which makes the coal rock, the mechanical structure and the hydraulic system interact with one another. The response differences of the caving mechanism under different rock materials are analyzed. The vibration and fault sensitive areas of the caving mechanism are determined. This study has a reference significance for improving the caving mechanism's structural design and failure prevention. The findings of the study provide guidance for a new intelligent coal rock identification technology based on vibration signals.

## Model establishment

### Mechanical–hydraulic co-theoretical model

The impact of coal rock on the caving mechanism is essentially the collision of two entities. The jack plays a vibration buffer role in the impact, similar to the spring system^20^. The caving mechanism can be simplified as a two-dimensional plane model^21, 22^. Based on hertz contact theory^23, 24^ and equivalent spring theory^25, 26^, the mechanical–hydraulic co-theoretical model of the caving mechanism impact by coal rock is established.

**Figure 2:**
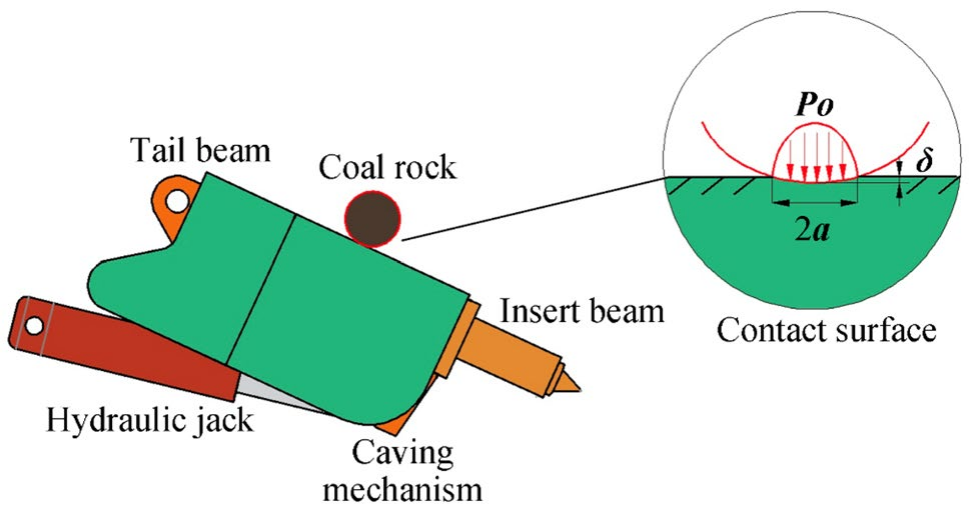
Schematic diagram of the caving mechanism under impact.

When the coal rock impacts the caving mechanism, a contact force is generated at the contact position, and a slight extrusion deformation occurs between them. The contact between the coal rock and the caving mechanism can be equivalent to the contact between a sphere and a plane in curvature^27, 28^. The contact form of the sphere and plane is shown in Fig. 2.

Normal extrusion deformation $\delta$ is generated at the contact position. The compressive stress distribution after contact is a semi-ellipsoid. The relationship between elastic contact force $F_{{\text{c}}}$ and deformation $\delta$ is as follows:

1 $$ F_{{\text{c}}} = \frac{4}{3}R_{0}^{\frac{1}{2}} E_{0} \delta^{n} $$

where $R_{0}$ is the equivalent radius, $E_{0}$ is the equivalent elastic modulus, $n$ is the stiffness coefficient. The nonlinear spring damping model based on Hertz contact theory considers the energy dissipation of two objects. The contact force is divided into an elastic force and a damping force:

2 $$ F_{{\text{c}}} = K_{{\text{c}}} \delta^{n} + D\delta_{{\text{r}}} $$

3 $$ K_{{\text{c}}} = \frac{4}{3}R_{0}^{\frac{1}{2}} E_{0} $$

where $K_{{\text{c}}}$ is the nonlinear contact stiffness, $\delta_{{\text{r}}}$ is the relative impact velocity, and the damping coefficient $D$ can be calculated as follows:

4 $$ D = \frac{3}{{4\delta_{{\text{r}}}^{{^{( - )} }} }}K_{{\text{c}}} (1 - e^{2} )\delta^{n} $$

where $\delta_{{\text{r}}}^{( - )}$ is the initial relative impact velocity. $e$ is the elastic restitution coefficient. The contact force between the coal rock and the control mechanism can be obtained as follows:

5 $$ F = K_{{\text{c}}} \delta^{n} \left[ {1{ + }\frac{{3(1 - e^{2} )\delta_{{\text{r}}} }}{{4\delta_{{\text{r}}}^{( - )} }}} \right] $$

The tail beam jack is the primary bearing component in the caving mechanism, and the hydraulic system can be considered the spring damping system. The equivalent stiffness of the jack is:

6 $$ K = \frac{{2S\alpha E_{{\text{s}}} }}{{S + 2L_{{\text{f}}} k_{{\text{f}}} \alpha E_{{\text{s}}} }} $$

where $S$ is the cross-sectional area of the liquid column, $k_{{\text{f}}}$ is the volume compressibility coefficient of the liquid, $L_{{\text{f}}}$ is the effective height of the liquid column, $\alpha$ is the cylinder wall thickness, and $E_{{\text{s}}}$ is the elastic modulus of the cylinder block.

**Figure 3:**
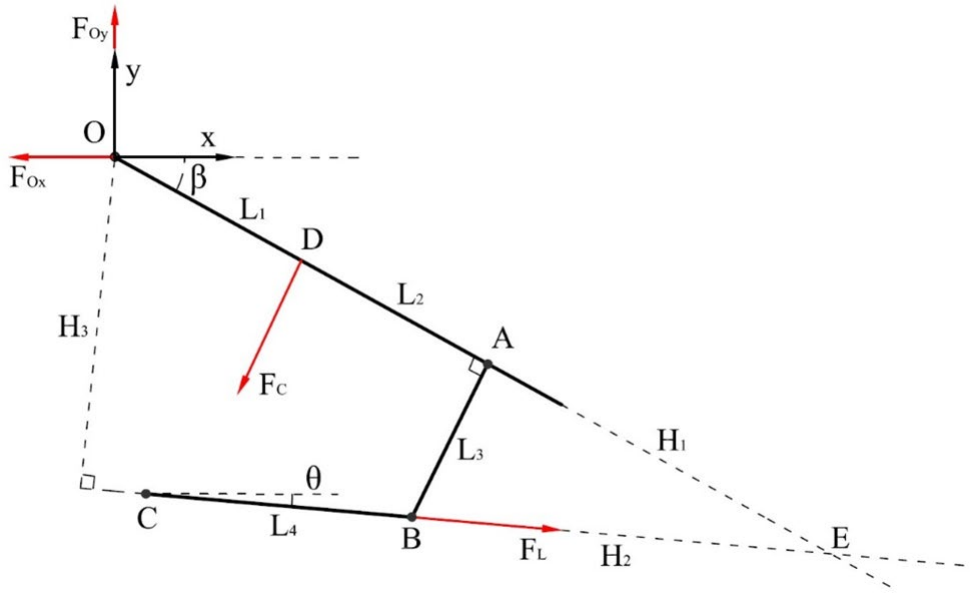
Force analysis diagram of the caving mechanism. O is the hinge point between the tail beam and shield beam, A is the vertical point between the upper plane of the tail beam and the lower hinge point of the tail beam, B is the hinge point between the tail beam and jack, C is the hinge point between jack and shield beam, D is the contact point between coal rock and tail beam, E is the intersection point between the jack and the upper plane of the tail beam, *β* is the included angle between tail beam and horizontal plane, and *θ* is the included angle between jack and horizontal plane.

From Fig. 3, according to the principle of force balance and moment balance^29^, it can be known that:

7 $$ \sum {F_{x} = 0,\sum {F_{y} = 0} } ,\quad \left\{ {\begin{array}{*{20}c} {F_{Oy} = F_{{\text{C}}} \cos \beta + F_{{\text{L}}} \sin \theta } \\ {F_{Ox} = F_{{\text{L}}} \cos \theta - F_{{\text{C}}} \sin \beta } \\ \end{array} } \right. $$

8 $$ \sum {M(O) = 0} ,\quad F_{{\text{L}}} H_{3} - F_{{\text{C}}} L_{1} = 0 $$

The trigonometric function and similar triangle theory are used to obtain the following:

9 $$ \left\{ {\begin{array}{*{20}c} {\sin (\beta - \theta ) = \frac{{L_{3} }}{{H_{2} }}} \\ {\tan (\beta - \theta ) = \frac{{L_{3} }}{{H_{1} }}} \\ {\frac{{L_{3} }}{{H_{3} }} = \frac{{H_{2} }}{{L_{1} + L_{2} + H_{1} }}} \\ \end{array} } \right. $$

Then, we obtain the relationship between elastic contact force $F_{C}$ and displacement change $x_{L}$ of the tail beam jack:

10 $$ F_{{\text{C}}} = \left( {1 + \frac{{L_{2} }}{{L_{1} }} + \frac{{L_{3} }}{{L_{1} \tan (\beta - \theta )}}} \right)\sin (\beta - \theta )\frac{{2S\alpha E_{{\text{s}}} }}{{S + 2L_{{\text{f}}} k_{{\text{f}}} \alpha E_{{\text{s}}} }}x_{{\text{L}}} $$

### Metal plate impacted by coal rock test bench

**Figure 4:**
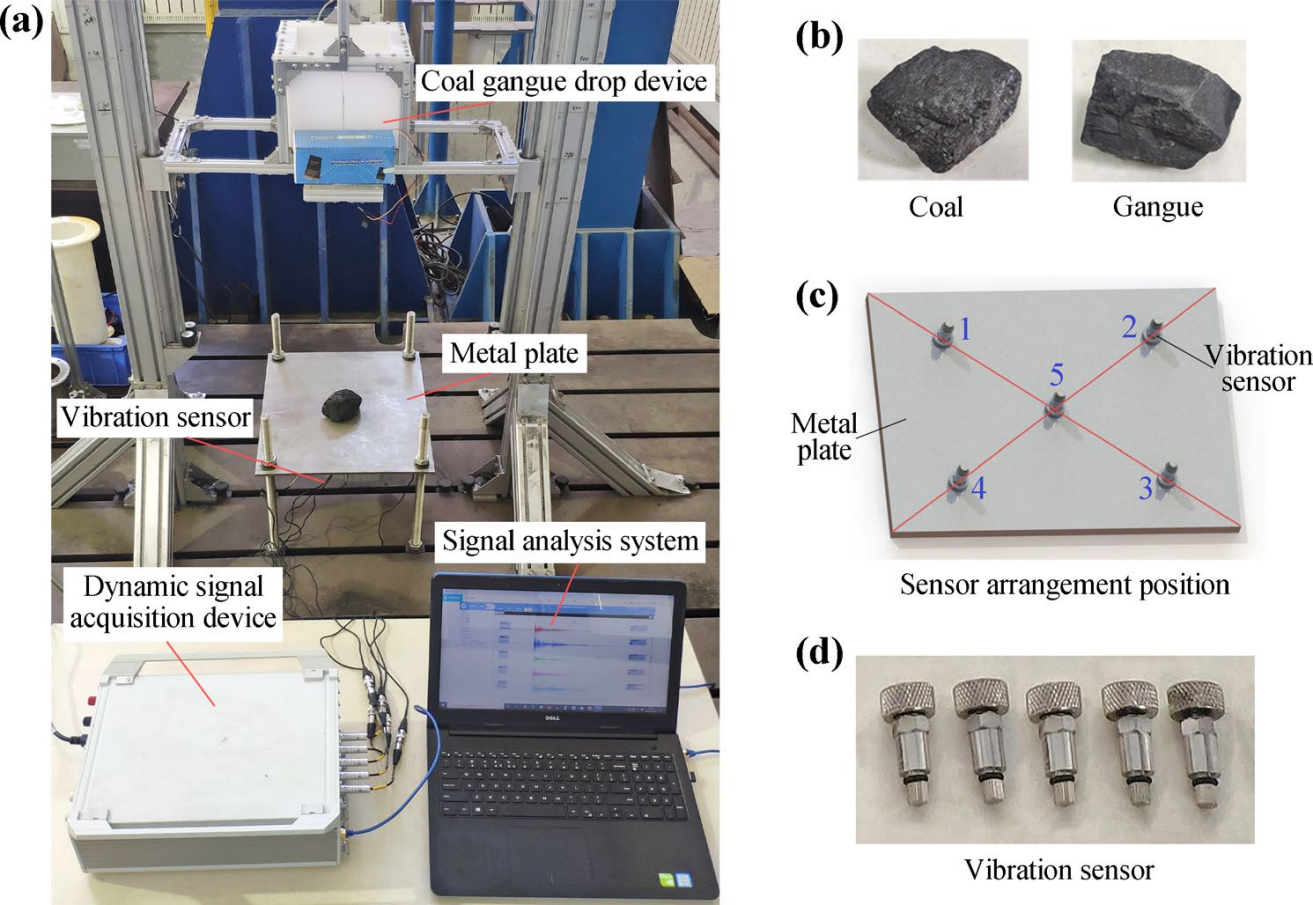
Metal plate impacted by coal rock test bench: (**a**) layout of the test bench, (**b**) types of coal rocks used in the test, (**c**) layout of sensors, and (**d**) acceleration vibration signal sensor.

The caving mechanism of top coal hydraulic support is mainly composed of metal plate welding. Since the contact part between the caving mechanism and the collapsed coal rock is a metal plate in the actual working condition, the caving mechanism can be simplified to a metal plate in the impact test^30^. In order to verify the difference in vibration response of the caving mechanism under the impact of different coal rock materials, the metal plate impacted by coal rock test bench was designed, as shown in Fig. 4a. The metal plate is fixed at all four corners and has five acceleration vibration sensors mounted on its bottom, as shown in Fig. 4c. The sensors used are 1A102E piezoelectric acceleration sensors from Jiangsu Donghua Testing Technology Co., Ltd., as shown in Fig. 4d. Sensors 1 to 4 are arranged on the four diagonal lines of the metal plate, and sensor 5 is arranged at the center of the metal plate. The coal rock is released through the coal gangue drop device. The released coal rock is coal and gangue respectively, as shown in Fig. 4b. The sensor signals are acquired by the DH8302 Dynamic signal acquisition device from Jiangsu Donghua Testing Technology Co., Ltd.

### Mechanical–hydraulic co-simulation model

**Figure 5:**
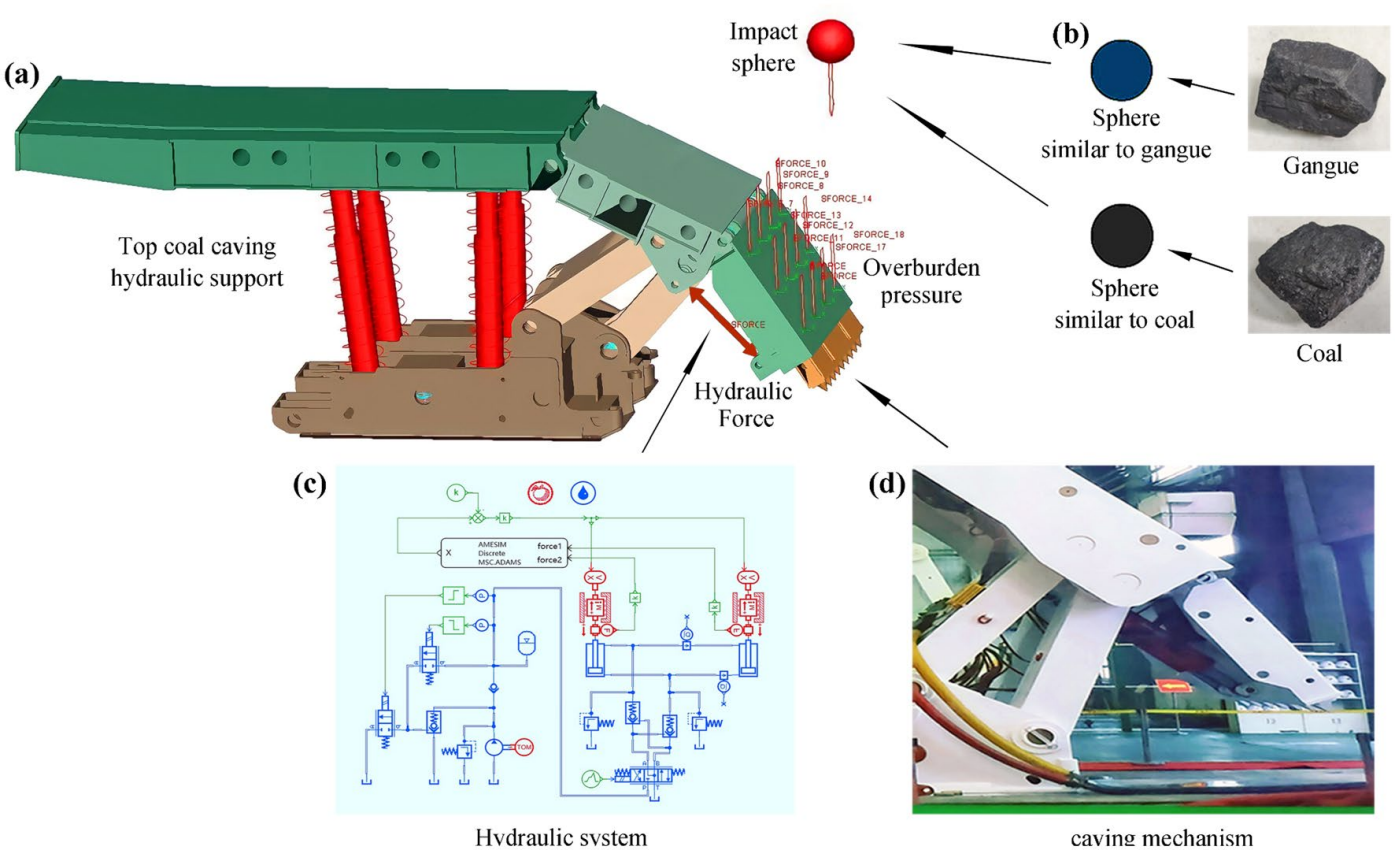
Mechanical–hydraulic co-simulation model. (**a**) The mechanical structure model of the hydraulic support. (**b**) Establish the impact sphere model based on real coal and gangue. (**c**) The hydraulic system provides the hydraulic force, controls the mechanism, and bears the impact load. (**d**) The actual caving mechanism.

Structural and hydraulic system design is performed for the hydraulic support. A multi-body dynamics model was built based on Adams^31^, as shown in Fig. 5a, and a hydraulic system model was built based on AMESim^32^, as shown in Fig. 5c. The co-simulation model is established according to the actual caving mechanism, which belongs to ZF5600/16.5/26 hydraulic support, as shown in Fig. 5d. Adams and AMESim can realize complex mechanical–hydraulic co-simulation by configuring the interface environment variables^33, 34^. In the co-simulation process, the second is divided into n steps. At 1/n second, after the simulation starts, the kinematics data is calculated by Adams and transmitted to AMESim through the co-simulation module. At 2/n second, AMESim calculates the hydraulic pressure based on the received data and passes the hydraulic data to Adams. Adams then calculates kinematic data based on the received hydraulic data and passes the new data again. And so on, to realize the collaborative calculation between the two software.

#### Multi-body dynamics model

**Figure 6:**
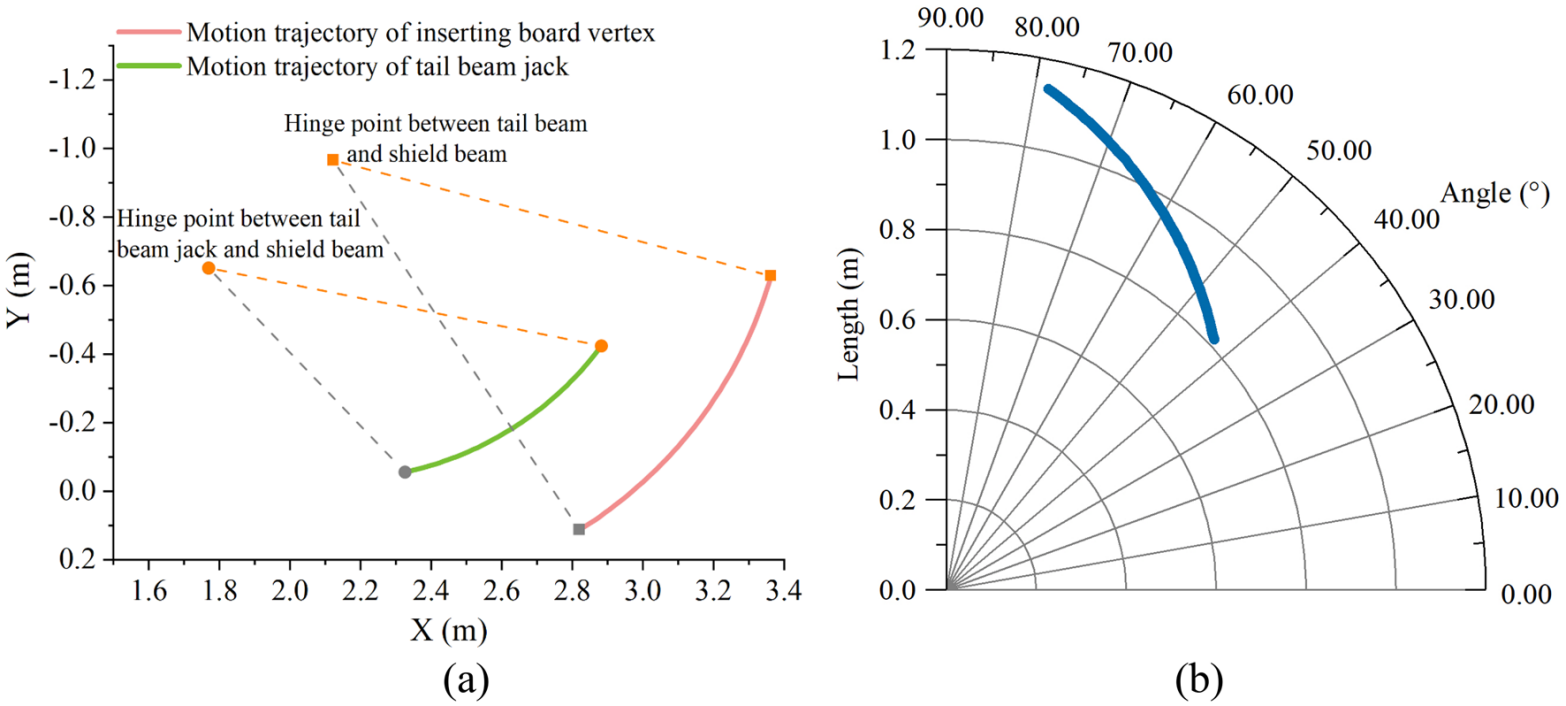
Component kinematic check: (**a**) Motion tracking of the caving mechanism; (**b**) Polar coordinate relationship between jack length and included angle.

In Adams, motion pairs are added for each component, motion drive is added for each jack, and no interference between components is confirmed through kinematic inspection^35, 36^. The hinge point between the tail beam jack and the tail beam is selected as the trajectory point of the tail beam jack. The endpoint of the tail beam is selected as the trajectory point of the tail beam. The angle between the tail beam jack and the vertical plane is used as the drawing reference angle. The motion track of the caving mechanism and the polar coordinate relationship between the jack length and included angle are shown in Fig. 6.

Figure 6a shows that the hinge joint of the jack piston rod moves from bottom to top, which drives the tail beam to swing upwards. The motion trace is an arc centered on the hinge joint of the tail beam and shield beam. Figure 6b shows that a longer extension length of the jack corresponds to a greater angle with the vertical plane. The tail beam of the caving mechanism has only one driving part: the tail beam jack. The extension length of the jack determines the posture of the tail beam. The established simulation process is consistent with the actual situation.

By adding multiple constant spatial forces, the uniform load was added above the caving mechanism to simulate the initial bearing capacity of coal rock to the control mechanism. The structural parameters of the jack are shown in Table 1. The impact load is applied to the caving mechanism through the free fall of coal rock, which mainly includes coal and gangue, as shown in Fig. 5b. The properties of the coal and gangue materials^37^ are shown in Table 2. The radius of the coal rock sphere is set to 80 mm. According to formula (3), the contact stiffness is calculated to be 1.426 × 10^9^ N/m. The contact damping is 0.1% of the contact stiffness. The Coulomb friction model is adopted for the friction between coal rock and the tail beam. Because there is dry friction between the coal rock and tail beam, the dynamic friction coefficient is set as 0.1, and the static friction coefficient is set as 0.15. In this simulation, the legs of the hydraulic support are only used as a support aid, and its support effect is similar to that of a spring, so the legs are simplified as an equivalent spring. According to formula (6), the equivalent spring stiffness is 1.766 × 10^9^ N/m. Thus, the mechanical structure model of the hydraulic support is finally established, as shown in Fig. 5a.

**Table 1:** Structural parameters of the jack.

Component	Diameter of the piston rod (m)	Diameter of the cylinder (m)	stroke (m)
tail beam jack	0.105	0.14	0.43

**Table 2:** Properties of steel and coal rock materials.

Material	Density (kg/m^3^)	Elastic modulus (N/m^2^)	Poisson ratio
Steel	7850	$2.06 \times 10^{11}$	0.3
Coal	1700	$3.5 \times 10^{9}$	0.28
Gangue	2800	$3.4 \times 10^{10}$	0.3

#### Hydraulic system model

In the AMESim hydraulic system^38^, the output end of the co-simulation module is connected to the pressure sensor of the jack, and the input end is connected to the displacement sensor of the jack. Therefore, through the co-simulation module, the displacement solution results of the Adams are transferred to AMESim in real-time, and the hydraulic pressure solution results of the AMESim are transferred to Adams in real-time. High water-based hydraulic oil with 95% water content is used in the hydraulic system. The temperature is set at 20 °C. A stop valve is added to the circuits of the jack to realize the two-way hydraulic lock. The hydraulic pump station is established by an accumulator, a constant-torque motor, a proportional valve, a unloading valve and other components. The unloading valve determines the maximum output pressure of the pumping station, which is typically designed for 31.5 MPa. Therefore, maximum output pressure of the pump station is set at 31.5 MPa. The reversing valve uses a Y-type three-position four-way valve for good braking performance. When the reversing valve is in the middle position, both chambers of the jack are tightly sealed, which avoids large displacement of the piston rod due to an external force. The piecewise linear signals are connected to the input of the reversing valve to control the valve opening, which controls the jack's movement and the caving mechanism's posture. The hydraulic system design is shown in Fig. 5c. The red part is the mechanical structure module, the blue part is the hydraulic system module, the green part is the signal transmission module, and the black part is the co-simulation module of Adams and AMESim.

## Results and discussion

### Experimental results of metal plate impacted by coal rock

Use coal and gangue with similar shapes and sizes to freely drop from the same height to impact the metal plate. The dumping position of coal and gangue is directly above the metal plate. The coal rock impact location is the center of the metal plate. After each impact experiment, the vibration response signals of 5 positions of the metal plate were collected by 5 sensors and DH8302 dynamic signal collector. Finally, the vibration response data of the metal plate is stored and extracted by the signal analysis system.

**Figure 7:**
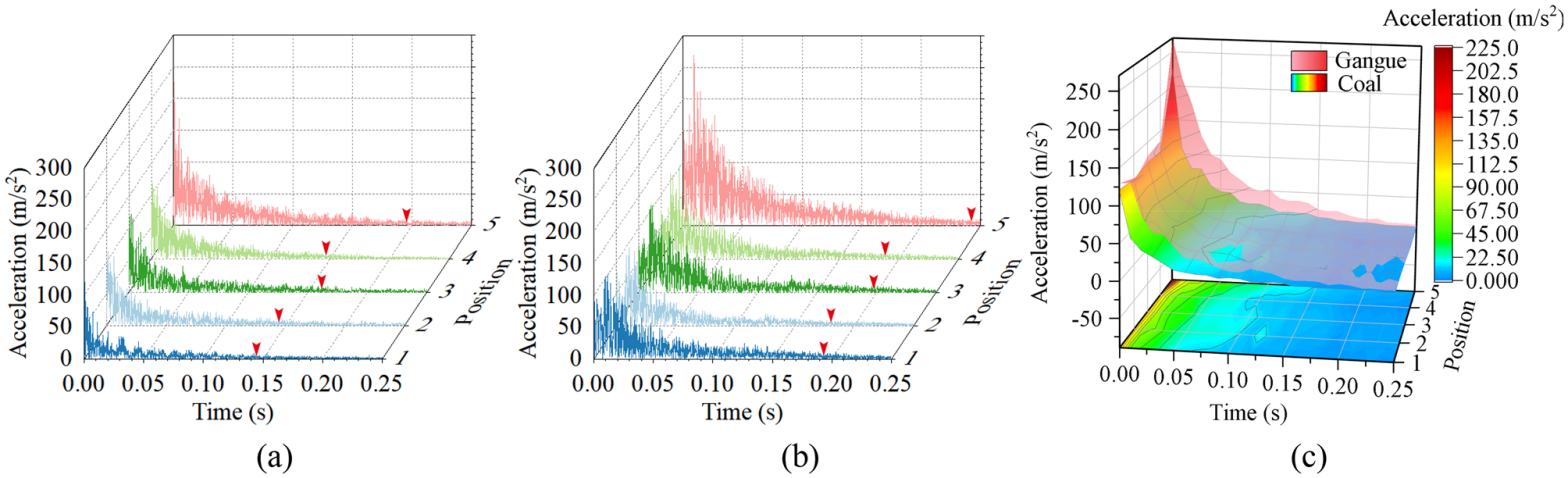
Response of metal plate under coal rock impact: (**a**) response of metal plate under coal impact, (**b**) response of metal plate under gangue impact, and (**c**) difference in impact response surface caused by coal and gangue.

The amplitude of the vibration signal describes the magnitude and strength of the vibration response of the metal plate. The response signal of the metal plate in this experiment is based on the symmetric vibration above and below the zero line. For effective comparison and analysis of the difference in vibration response caused by coal and gangue, only the absolute value of the vibration signal is retained. The vibration responses of the metal plate under the impact of coal and gangue are shown in Fig. 7a,b, respectively. Finally, the amplitude of the vibration response signal is extracted, and the five groups of coal-induced vibration signals are combined into a coal impact response surface. The five groups of gangue-induced vibration signals are combined into a gangue impact response surface. The difference between the two response surfaces is shown in Fig. 7c. The difference refers to the deviation distance between two impact response surfaces. The graphical distribution on the basal plane is the projection of the coal impact response surface onto the time-position plane.

From Fig. 7a,b, the metal plate's vibration response is similar at positions 1–4, and the vibration response is greatest at position 5. That is, the closer to the point of impact, the stronger the vibration response of the metal plate. The amplitude of the impact response is similar for the position with a similar distance from the point of impact. The response of metal plate under coal rock impact shows the maximum amplitude at the moment of impact, and then decreases uniformly with time until it is smooth. The vibration time of the metal plate is very short. When the vibration amplitude is less than 10 m/s^2^, it is considered as steady state and is marked by a red triangle. The vibration time of the metal plate at positions 1–4 is shorter than that of position 5. Taking position 5 as an example, the vibration stabilization time of metal plate under the impact of coal is 0.193 s, and that under the impact of gangue is 0.241 s. It can be obtained that the vibration time of the metal plate under gangue impact is greater than under coal impact. The closer to the impact position, the longer the metal plate vibration time.

From Fig. 7c, it can be seen that the vibration response of the metal plate under gangue impact is greater than under coal impact. The difference in vibration response of metal plate caused by gangue and coal is maximum at the moment of impact, and decreases with time afterward. The difference in vibration response of the metal plate at positions 1–4 is similar, and the difference in vibration response is the largest at position 5. It can be obtained that the metal plate has a significant differential vibration response to coal and gangue at the position close to the impact. Therefore, there are differences in the vibration response of metal plate under the impact of different coal rock materials. The caving mechanism is made of metal plate welding. Hence, it is feasible to study the impact vibration response of the caving mechanism in more detail based on the mechanical–hydraulic coupling simulation.

### Simulation results of caving mechanism impacted by coal rock

**Figure 8:**
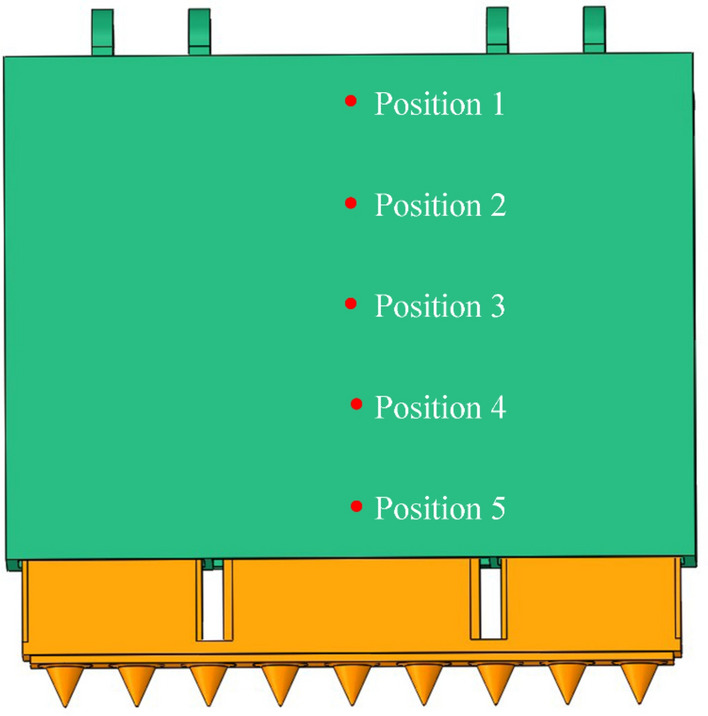
Impact position of the caving mechanism.

Due to the different properties of coal and gangue materials, coal and gangue impacting the caving mechanism must cause different responses. This differential response is the basis for coal rock identification in intelligent mining. Based on the above research, the response difference of the caving mechanism impacted by coal and gangue is analyzed with the variables of material, position, volume and velocity. The two materials of coal and gangue are shown in Table 2 above. The five impact positions are evenly distributed along the center line of the tail beam, as shown in Fig. 8. The coal and gangue radii are set as 0.05 m, 0.06 m, 0.07 m, 0.08 m, 0.09 m and 0.10 m. The interval is 0.01 m on average. The impact velocity is determined by the caving height, which is set as 0.5 m, 0.7 m, 0.9 m, 1.1 m, 1.3 m and 1.5 m. The interval is 0.2 m on average. The corresponding velocities are 3.12 m/s, 3.70 m/s, 4.20 m/s, 4.64 m/s, 5.04 m/s and 5.42 m/s. When controlling variables, select the middle value as the default value, such as a radius of 0.08 m and velocity of 4.20 m/s. By controlling the variables, 360 groups of coal and gangue impact simulations are performed, and the response values of the force, hydraulic pressure and movement parameters of the mechanism are extracted. The mechanical structure vibration signals of the caving mechanism are collected at the position of the hinge point between the tail beam and the jack, which are the displacement of the tail beam, velocity of the tail beam, and acceleration of the tail beam. The hydraulic system vibration signals of the caving mechanism are collected from the jack, which are the pressure in rodless cavity, pressure in rod cavity, and support force of the jack.

#### Response difference of caving mechanism at different impact positions

**Figure 9:**
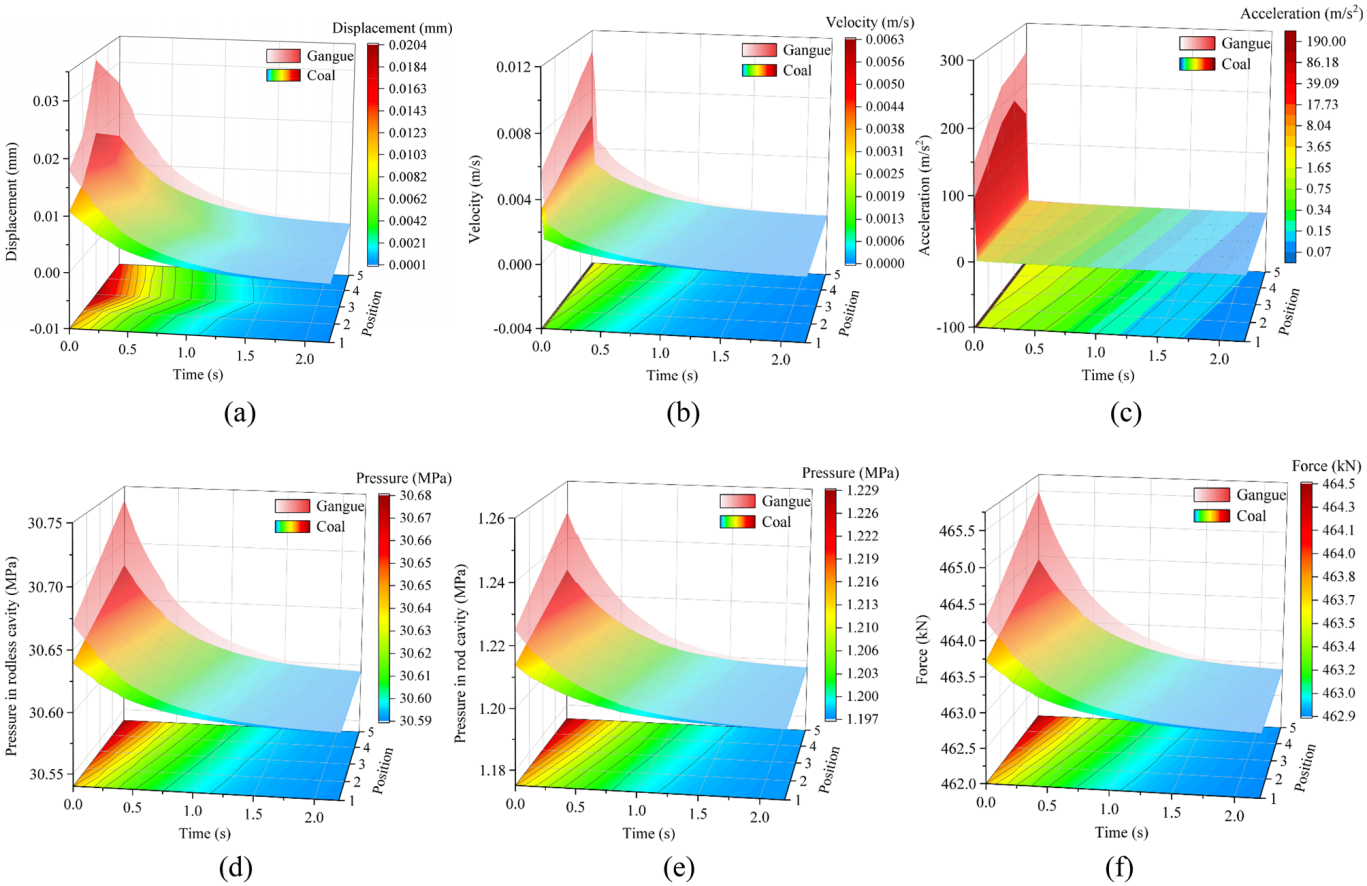
Response difference of caving mechanism at different impact positions: (**a**) displacement of the tail beam, (**b**) velocity of the tail beam, (**c**) acceleration of the tail beam, (**d**) pressure in rodless cavity, (**e**) pressure in rod cavity, and (**f**) support force of the jack.

By changing the impact position of the caving mechanism, we plot the response surfaces at different impact positions with the impact instant as 0 s, as shown in Fig. 9. The graphical distribution on the basal plane is the projection of the coal impact response surface onto the time-position plane.

From Fig. 9, the vibration response of the caving mechanism caused by gangue is greater than that caused by coal. A lower position of the caving mechanism corresponds to a greater vibration response. The difference between coal-induced and gangue-induced vibration response is greatest at the instant of impact and decreases with time thereafter. The impact position is closer to the end of the caving mechanism, the difference in vibration response between coal-induced and gangue-induced increases. Among them, the left end opening of the two surfaces of displacement of the tail beam, pressure in rodless cavity, pressure in rod cavity and support force of the jack are larger, that is, the vibration response difference between coal-induced and gangue-induced in these areas are obvious. The left end opening of the two surfaces of the velocity of the tail beam and acceleration of the tail beam are small, that is, the vibration response difference between coal-induced and gangue-induced in these areas are not obvious or only at the instant of impact. The most obvious vibration response is when impacting the end of the caving mechanism (position 5). Thus, position 5 is most suitable for collecting coal rock identification vibration signals. This position will also likely fail due to the coal rock impact.

#### Response difference of caving mechanism under different impact velocities of rock

**Figure 10:**
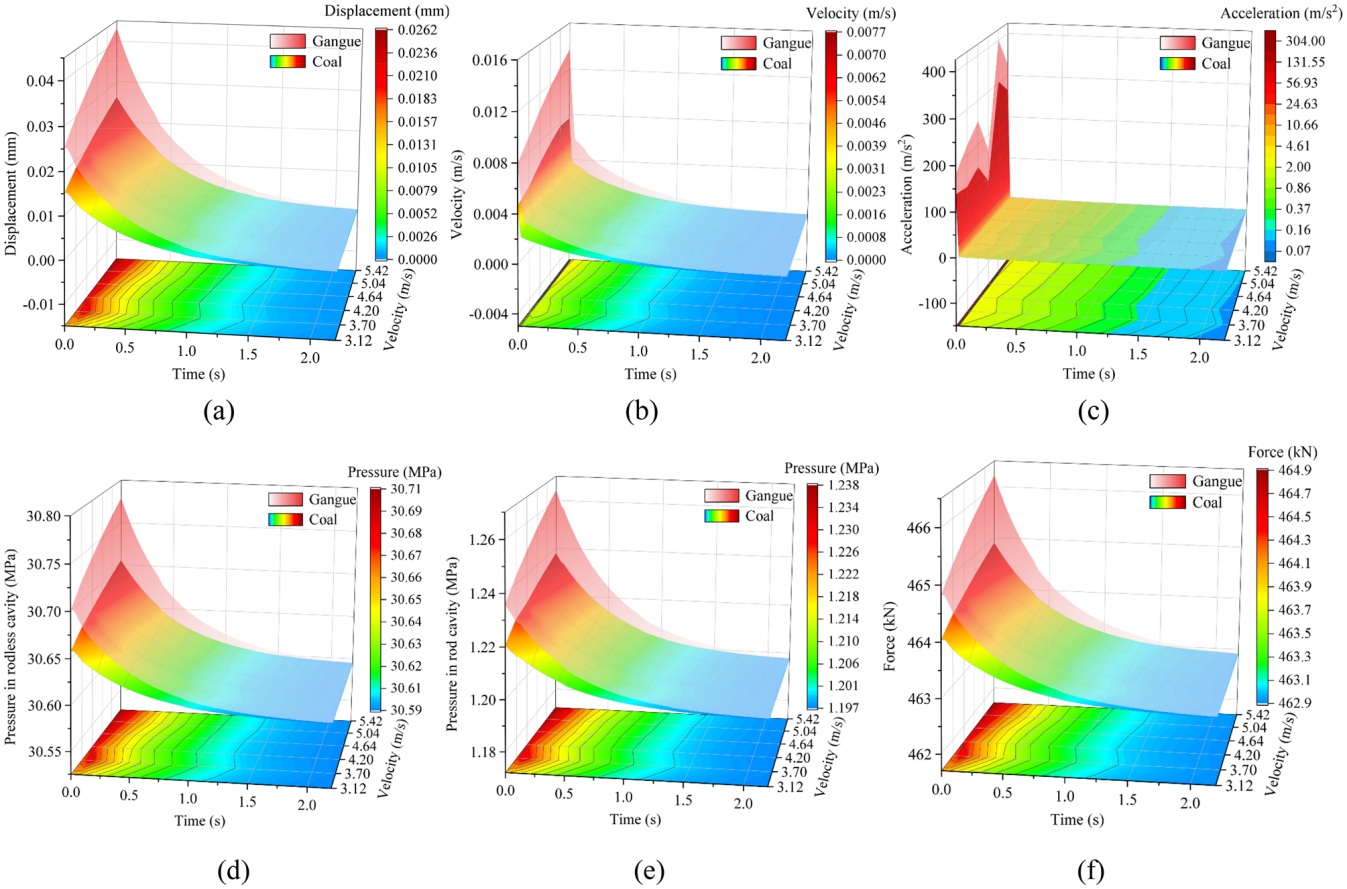
Response difference of caving mechanism under different impact velocities of rock: (**a**) displacement of the tail beam, (**b**) velocity of the tail beam, (**c**) acceleration of the tail beam, (**d**) pressure in rodless cavity, (**e**) pressure in rod cavity, and (**f**) support force of the jack.

Based on the above research, position 5 is the most sensitive to coal rock impact. Therefore, the impact position is set to position 5. Take the impact instant as 0 s and plot the response surfaces under different impact velocities, as shown in Fig. 10.

From Fig. 10, the vibration response of the caving mechanism caused by gangue is greater than that caused by coal. The difference between coal-induced and gangue-induced vibration response is greatest at the instant of impact and decreases with time thereafter. The higher the impact velocity, the greater the vibration response of the caving mechanism. A higher impact velocity corresponds to a more noticeable response difference between coal-induced and gangue-induced. Among them, the left end opening of the two surfaces of displacement of the tail beam, pressure in rodless cavity, pressure in rod cavity and support force of the jack are larger, that is, the vibration response difference between coal-induced and gangue-induced in these areas are obvious. The left end opening of the two surfaces of the velocity of the tail beam and acceleration of the tail beam are small, that is, the vibration response difference between coal-induced and gangue-induced in these areas are not obvious or only at the instant of impact. When the impact velocity is high, there is the most obvious vibration response of the caving mechanism. Thus, the high-velocity impact is more suitable for identifying coal rock based on vibration signal and is most likely to damage the caving mechanism.

#### Response difference of caving mechanism under impact of different volumes of rock

**Figure 11:**
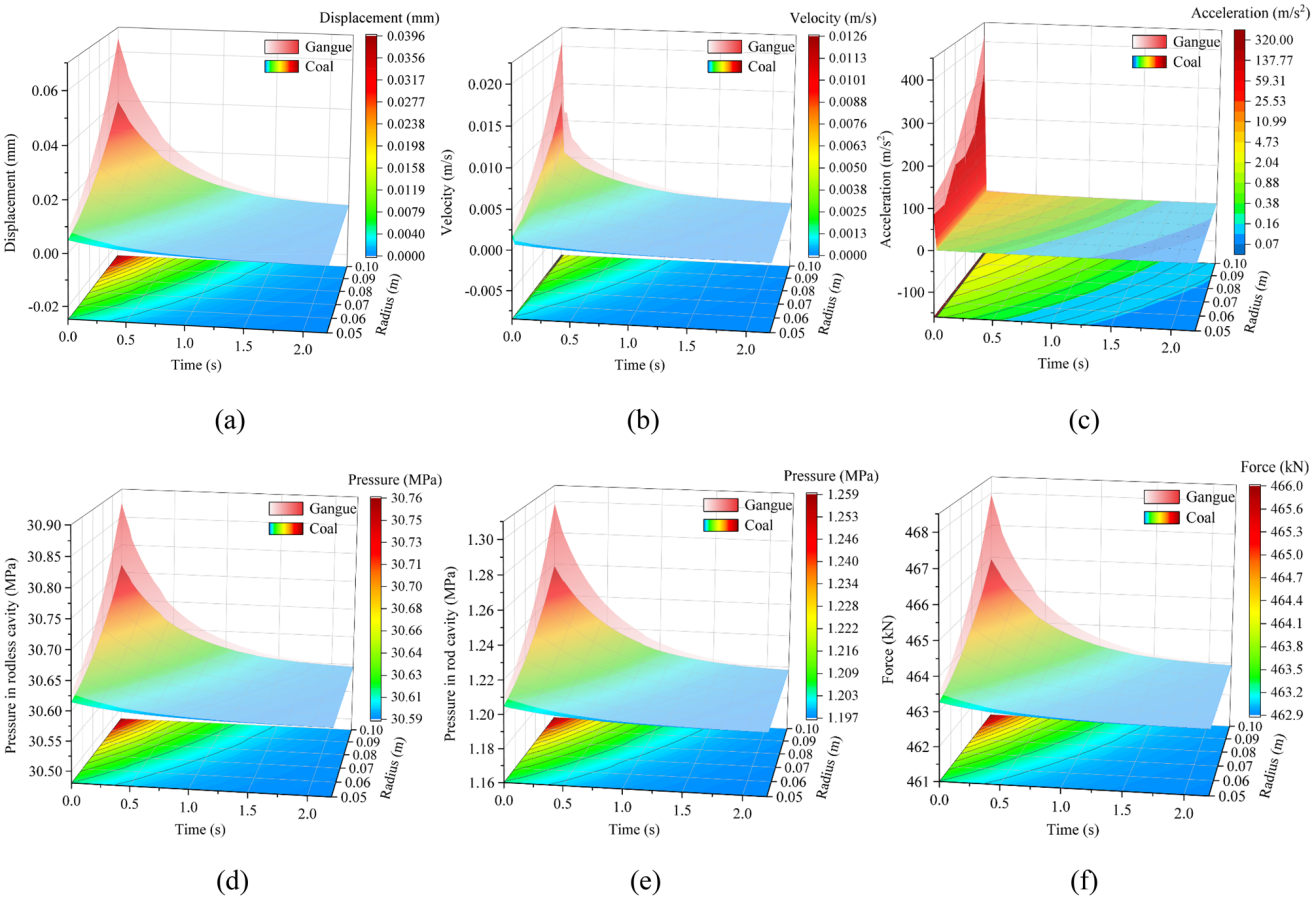
Response difference of caving mechanism under the impact of different volumes of rock: (**a**) displacement of the tail beam, (**b**) velocity of the tail beam, (**c**) acceleration of the tail beam, (**d**) pressure in rodless cavity, (**e**) pressure in rod cavity, and (**f**) support force of the jack.

Based on the above research, the impact position is set to position 5. We take the impact instant as 0 s and plot the response surfaces under different coal rock volumes, as shown in Fig. 11.

From Fig. 11, the larger the volume of coal rock, the stronger the impact response of the caving mechanism. A larger volume of coal rock corresponds to a more noticeable response difference between coal-induced and gangue-induced. Compared with the above two groups, the left end openings of the two surfaces of the six parameters are all small. Especially when the volume of coal rock is small, the upper and lower surfaces are nearly coincident. Thus, the influence of rock volume parameters on the response difference of the caving mechanism by coal and gangue impact is weak. The large-volume coal rock can be used for coal rock identification research, and it is easier to damage the caving mechanism. The small-volume rock is not suitable for coal rock identification research.

The above results showed that when the position of the caving mechanism is lower, the velocity of coal rock is higher, and the volume of coal rock is larger, the vibration response difference of the caving mechanism is more pronounced. The impact position and impact velocity significantly affect the response difference, while the coal rock volume weakly affects the response difference. The vibration response of pressure in rodless cavity, pressure in rod cavity, jack support force and tail beam displacement are more prominent, while the vibration response of tail beam velocity and tail beam acceleration are weak. Under the same conditions, the impact of gangue can cause a greater vibration response in the caving mechanism than the impact of coal. The most effective vibration response data of the caving mechanism can be obtained by reasonably selecting the impact position near the end of the tail beam and adopting the higher impact velocity.

## Conclusions

In this paper, the metal plate has different vibration responses under the impact of coal rock of different materials is verified experimentally. After that, the numerical analysis model and mechanical–hydraulic co-simulation model of the caving mechanism are established, making the coal rock, the mechanical structure, and the hydraulic system interact. The vibration response difference of the caving mechanism under coal rock impact is analyzed under different rock materials, rock volume, impact velocity and impact position. The conclusions are as follows:

1. The vibration response of the metal plate under gangue impact is greater than that under coal impact. The metal plate has a noticeable difference in vibration response to coal and gangue near the impact position.
2. The vibration response of the caving mechanism under the impact of coal and gangue is different. The impact of gangue causes a greater vibration than the impact of coal. The lower the impacted position, the higher the impact velocity, and the larger the coal rock volume, the more pronounced the difference in response.
3. The response difference is greatly influenced by the impact position and velocity but less by the coal rock volume. The response difference is obvious in the parameters of displacement of the tail beam, pressure in rodless cavity, pressure in rod cavity and support force of the jack but weak in the parameters of velocity and acceleration of the tail beam.
4. The high impact velocity and large volume of coal rock are more suitable for collecting coal rock identification vibration signals and are most likely to damage the caving mechanism.
5. The end of the tail beam is the vibration-sensitive area of the caving mechanism. In the actual working process, extra attention should be paid to protecting the end of the tail beam. In the hypothetical case, the degree of pre-crushing of the top coal is increased as much as possible, and the failure probability of the caving mechanism can be reduced by reducing the velocity of the caving and the size of the broken top coal.

This study has a reference significance for improving the caving mechanism's structural design and failure prevention. The findings of the study provide guidance for a new intelligent coal rock identification technology based on vibration signals. This study does not consider the caving mechanism's vibration response in the lifting and downswing process. Therefore, the mechanical–hydraulic coupling model can consider the motion state of the caving mechanism in further research.

### Ethics approval

The authors warrant that the work has not been published before in any form and that the work is not concurrently submitted to another publication. The authors also warrant that the work does not libel anyone, infringe anyone’s copyright, or otherwise violate anyone’s statutory or common law rights.

## Data Availability

The data that support the findings of this study are available from the corresponding author, Qingliang Zeng, upon reasonable request.
